# Analysis of Health System Size and Variability in Diabetic Eye Disease Screening in Wisconsin

**DOI:** 10.1001/jamanetworkopen.2021.43937

**Published:** 2022-01-18

**Authors:** Loren J. Lock, Mark Banghart, Roomasa Channa, Maureen A. Smith, Meghan B. Brennan, Alejandra Torres Diaz, Yao Liu

**Affiliations:** 1 Department of Ophthalmology and Visual Sciences, School of Medicine and Public Health, University of Wisconsin, Madison; 2 Department of Population Health Sciences, School of Medicine and Public Health, University of Wisconsin, Madison; 3 Department of Family Medicine and Community Health, School of Medicine and Public Health, University of Wisconsin, Madison; 4 Department of Medicine, School of Medicine and Public Health, University of Wisconsin, Madison

## Abstract

This cohort study uses statewide claims data to assess variability in diabetic eye disease screening across Wisconsin health systems and to examine the association between patient health system and screening receipt.

## Introduction

Diabetic eye disease remains the leading cause of blindness in the US, largely because of low screening rates.^1,2,3^ Previous studies have tended to focus on patient-level factors associated with screening.^2^ Thus, interventions have emphasized patient education, yielding only modest improvements.^4^ In this study, we used an all-payer, statewide claims database to assess screening variability across health systems and to determine whether a patient’s health system may be associated with screening receipt.

## Methods

This retrospective cohort study was deemed exempt from review by the University of Wisconsin Health Sciences Institutional Review Board and informed consent was waived because we conducted secondary analysis of a deidentified data set. All research activities were conducted in accordance with the Declaration of Helsinki. The study followed the Strengthening the Reporting of Observational Studies in Epidemiology (STROBE) reporting guideline.

We analyzed deidentified data from the Wisconsin Health Information Organization All-Payer Claims Database, covering 75% of Wisconsin residents. We included adults (aged 18-75 years on the start date of the measurement period) who had primary medical insurance coverage throughout the baseline (October 2012 through September 2013) and measurement (October 2013 through September 2015) years and had been diagnosed with diabetes according to a validated claims definition.^5^ Patients received guideline-concordant screening^6^ if they had a claim billed for an examination with an eye care provider or telemedicine-based retinal imaging during the measurement year. Patient-level factors included age, sex, hierarchical condition category risk score, and primary care clinic rurality. Primary care clinic rurality was a surrogate measure of patient rurality because we did not have access to patient home zip codes.

We created multivariable logistic regression models to assess potential factors associated with screening receipt. We included the 143 health systems as 1 categorical variable with 101 possible values—that is, the largest 100 health systems plus the 43 smallest health systems aggregated and analyzed as a single health system. A health system comprised an affiliated group of physicians and/or clinics and was assigned based on a patient’s primary care provider. We used the absolute value of the odds ratio (OR) for each health system (using the health system with the median screening rate as the reference) to quantify the distribution of the effect of health system on screening receipt. Statistical analyses were performed from 2020 to 2021 using SAS version 9.4 (SAS Institute).

## Results

We included 119 347 adults with diabetes from 698 primary care clinics in 143 Wisconsin health systems. Most patients (74.4%) were older than 55 years (mean [SD] age, 60.9 [11.3] years), and 48.7% were women. The most common insurers were Medicare (58.4%), commercial (30.9%), and Medicaid (10.1%), and 18.6% of patients obtained care at a rural primary care clinic. There were 20 049 excluded patients; they were slightly younger; were more likely to be women, to be non-Medicare insured, and to obtain care from rural primary care clinics; and were less likely to have received eye screening.

Eye screening varied widely from 31.8% to 73.0%. There was less screening at smaller health systems (ie, those with smaller patient populations), as indicated by the smaller bubbles in deciles 1 and 2 in the Figure. The median magnitude of the effect of health system on the odds of screening receipt was 1.24 (IQR, 1.11-1.48). When we excluded health system from the model, patients who obtained care from rural vs urban primary care clinics were more likely to obtain screening (OR, 1.14 [95% CI, 1.11-1.18]; Table). However, this association reversed when health system was included (OR, 0.94 [95% CI, 0.91-0.98]).

**Figure.**
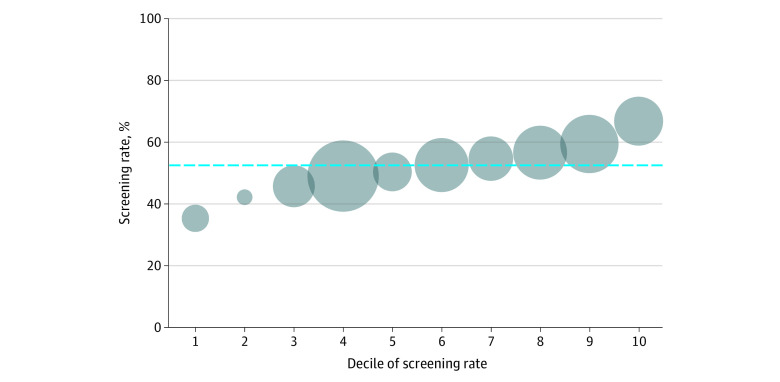
Variation in Diabetic Eye Disease Screening Rate Among the 100 Largest Health Systems in Wisconsin Bubble sizes correspond to the size of the patient population served by the 10 health systems within each decile of screening. The dashed blue line denotes the mean overall screening rate of 53.6%.

**Table.** Multivariable Analysis of Factors Associated With Diabetic Eye Disease Screening Receipt Among 119 347 Adults in Wisconsin

Characteristic	OR (95% CI)	
	Including health system	Excluding health system
Age, y		
≤54	1 [Reference]	1 [Reference]
55-64	1.33 (1.29-1.38)	1.33 (1.29-1.38)
65-75	2.02 (1.95-2.09)	2.03 (1.95-2.10)
Sex		
Men	1 [Reference]	1 [Reference]
Women	1.25 (1.22-1.28)	1.24 (1.21-1.27)
Insurance payer		
Commercial	1 [Reference]	1 [Reference]
Medicare	1.26 (1.22-1.30)	1.16 (1.12-1.19)
Medicaid	1.13 (1.08-1.19)	0.97 (0.93-1.02)
Other	0.93 (0.80-1.09)	1.14 (0.98-1.32)
Primary care clinic rurality		
Urban	1 [Reference]	1 [Reference]
Rural	0.94 (0.91-0.98)	1.14 (1.11-1.18)
HCC index	1.05 (1.04-1.07)	1.04 (1.03-1.05)

Abbreviations: HCC, hierarchical condition category; OR, odds ratio.

## Discussion

The results of this cohort study suggest that health system may be an important factor in diabetic eye disease screening receipt. Health system had a mean effect equivalent to that of sex, which is a well-known factor in diabetic eye disease screening receipt.^2^ We also found substantial variability in screening receipt, with less screening among patients obtaining care from smaller health systems and rural primary care clinics.

A limitation of this study is that the excluded patients could have biased our results, although their differences were slight. Interventions targeting health systems and rural primary care clinics may be important for increasing diabetic eye disease screening rates.
